# In vitro assessment of internal implant-abutment connections with different cone angles under static loading using synchrotron-based radiation

**DOI:** 10.1186/s12903-024-04156-2

**Published:** 2024-03-28

**Authors:** Johannes Angermair, Gerhard Iglhaut, Konrad Meyenberg, Wolfram Wiest, Alexander Rack, Simon Zabler, Tobias Fretwurst, Katja Nelson, Florian Kernen

**Affiliations:** 1 Private Practice for Oral and Maxillofacial Surgery, Wiesbaden, Germany; 2 Department of Oral and Maxillofacial Surgery, Translational Implantology, Medical Center, Faculty of Medicine, University of Freiburg, Hugstetter Strasse 55, 79106 Freiburg, Germany; 3 Private Office, Zurich, Switzerland; 4 Chair of X-ray Microscopy LRM, University Würzburg, 97074 Würzburg, Germany; 5 Experiments Division, ESRF – The European Synchrotron, Grenoble, France; 6 Deggendorf Institute of Technology DIT, Dieter-Görlitz-Platz 2, 94469 Deggendorf, Germany

**Keywords:** Dental implants, Implant design, Microgap formation, Mechanical testing, Synchrotron radiation

## Abstract

**Background:**

The stability of implant-abutment connection is crucial to minimize mechanical and biological complications. Therefore, an assessment of the microgap behavior and abutment displacement in different implant-abutment designs was performed.

**Methods:**

Four implant systems were tested, three with a conical implant-abutment connection based on friction fit and a cone angle < 12 ° (Medentika, Medentis, NobelActive) and a system with an angulated connection (< 40°) (Semados). In different static loading conditions (30 *N* − 90º, 100 *N* − 90º, 200 *N* − 30º) the microgap and abutment displacement was evaluated using synchrotron-based microtomography and phase-contrast radioscopy with numerical forward simulation of the optical Fresnel propagation yielding an accuracy down to 0.1 μm.

**Results:**

Microgaps were present in all implant systems prior to loading (0.15–9 μm). Values increased with mounting force and angle up to 40.5 μm at an off axis loading of 100 N in a 90° angle.

**Conclusions:**

In contrast to the implant-abutment connection with a large cone angle (45°), the conical connections based on a friction fit (small cone angles with < 12°) demonstrated an abutment displacement which resulted in a deformation of the outer implant wall. The design of the implant-abutment connection seems to be crucial for the force distribution on the implant wall which might influence peri-implant bone stability.

## Background

Dental implants are an established therapeutic option in modern dentistry demonstrating high success and survival rates in long-term studies [1]. Two-piece implants in which the implant and the abutment are screw-tightened are the most common form [2]. The connecting zone between implant and abutment, the implant-abutment connection (IAC) has to withstand multiaxial forces during masticatory function [3]. In principle, two IAC designs can be distinguished: Conical connections with interference fit (press-fit) and butt-joint connections with clearance fit of the two components [4]. The mechanical properties of press-fit conical connections are characterized by the angle of the connecting surfaces and the length of the mating zone which determine the amount of friction between the two manufactured parts [5]. This friction force is used in conical implant-abutment connections to ensure mechanical stability of the implant abutment complex [6]. In these connections the tightening of the abutment screw with the system specific torque value results in a defined axial displacement of the abutment into the implant which is responsible for the interference fit at the conical implant abutment interface [7]. Butt-joint connections, due to their horizontal or wide angled mating zone, lack friction fit, thus torque tightening of the abutment screw is the crucial element for vertical stabilization of the screw joint [8]. The discrimination between external and internal IAC´s is based on the position of the antirotational index: in internal connections the index is inside the implant body and in external connections outside of the body implying that butt-joint and conical connections can be internal connections, whereas external connections can only be a butt-joint connection [9]. The microgap within the IAC has been described to decrease in vitro after cyclic loading for both butt-joint and conical connection and is accompanied by an intrusion of the abutment into the implant body in conical connections [10–12]. However, there is no evidence for a characteristic failure mode of a certain type of implant abutment design. Mechanical complications such as abutment or screw loosening and ceramic chipping occur up to 4.1, and 11.6% respectively within the first 5 years independent of the design of the implant-abutment connection [13–15]. Furthermore, biological complications are discussed to originate due to the microgap formation along the implant and the abutment of the IAC. It has been hypothesized that the resulting microleakage could cause a bacterial colonization of the IAC and be a factor for peri-implant mucositis and peri-implantitis [16–20] with a mean prevalence of 43% and 22%, respectively 21]. To date the genesis of this multifactorial disease is not completely understood [22]. In recent studies titanium particles in the peri-implant tissue are discussed to influence the peri-implant inflammatory reaction [23]. These particles could originate from the IAC since abutment micromovement under cyclic loading results in wear in butt-joint and conical IACs. Wear particles found in the IAC show the same composition as particles found in the soft and hard peri-implantitis tissue [11, 24–26]. To ensure long-term prognosis and durability of dental implants mechanical testing is mandatory [11, 27, 28]. Therefore, load-to-failure and load-bearing capacity testing is performed using a standardized testing environment, the ISO Standard 14801:2008 [29]. To observe microgap formation under off axis load, X-ray radioscopy would be the method of choice, since it features excellent contrast between metal and air and applies in transmission. However, the moderate resolving power of clinical and industrial X-ray devices sets the limit of detectability of IAC microgaps to approx. 20 μm opening [30]. By replacing the X-ray anode in the setup with a particle accelerator (a synchrotron), this limit is easily overcome. Synchrotron facilities provide approx. 40 extremely bright X-ray beams simultaneously, continuously for a variety of experimental purposes among which feature imaging stations comprising radioscopy and micro tomography setups.

Using synchrotron-based radiation (SRX) allows to observe IAC microgaps down to sizes of 0.1 μm with unparalleled contrast and speed [31, 32]. Unlike clinical or industrial X-ray scanners which feature cone beams, SRX features quasi parallel beams. Therefore, for realizing high spatial resolution there is no need to downsize the object for setting it right in front of the X-ray anode. Instead, hard x-rays are penetrating large objects while directly imaging sub micrometer voids and cracks even in dense and highly reductive material [33]. Increasing the distance between object and detector does not affect exposure time but activates phase contrast through the mechanism of optical Fresnel-propagation thus increasing the detectability of microscopic cracks and voids (and microgaps) tenfold. Studies using SRX demonstrated the existence of the microgap without load in conical IAC and a change of the size of the microgap under load application [11, 12, 34, 35]. Besides the existence of a ubiquitous microgap a plastic deformation of the implant wall under loading has been described [12, 36]. The deformation of the implant shoulder due to abutment displacement and the mode of force distribution might induce stress on the surrounding bone, which could impair long-term stability [37, 38]. Based on mechanical considerations the mode of abutment displacement seems to depend on geometric parameters of the IAC [39]. 

Microgap behavior in conical implant-abutment-connections has been studied using SRX, there is no literature concerning the mode of displacement of the abutment in association with the cone angle and microgap formation. Therefore, the aim of this study was to quantitatively assess and compare microgap behavior and the mode of displacement in different implant-abutment designs using high resolution synchrotron-based microtomography. For the first time an implant system using a butt-joint connection was assessed using SRX at micrometer dimension to quantitatively describe and compare microgap formation in regard to the nature of the mating zone.

## Methods

### Sample preparation

**Fig. 1:**
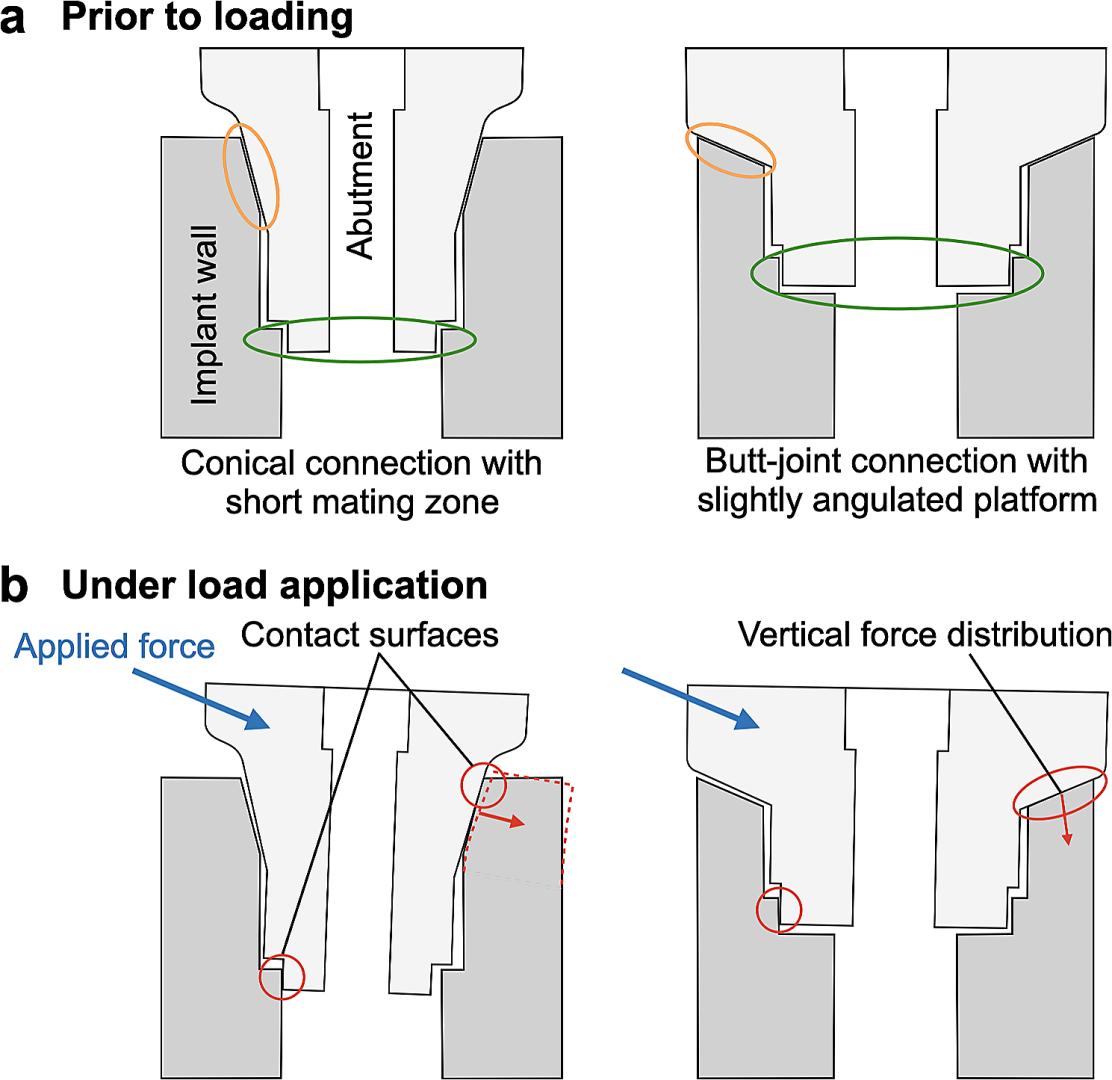
Schematic figure of conical connection with a short mating zone (mating zone: orange circle) and butt-joint connection with a slightly angulated platform before (**a**) and after load application (**b**). The antirotational indices are encircled in green color. Red lines demonstrate lateral force transmission on the implant wall in conical connections and vertical distribution in butt-joint connections. Butt-joint connections with an adequate preload of the abutment screw distribute the force more vertically into the implant shoulder

Two dental implants of three different implant systems with conical IACs and of one system with a butt-joint connection were tested in this study in order to evaluate intra-system variations as well as inter-system differences in the mechanical behavior of the system with regards to microgap formation at the IAC (Fig. 1a). The latter was measured in vitro by synchrotron phase-contrast radioscopy (PCR). The implants were: Medentika (MA, Microcone, RI 3.5 × 11 mm, Medentika GmbH, Huegelsheim, Germany), Medentis (MS, ICX-Templant, 4.1 × 12,5 mm, Medentis Medical GmbH, Ahrweiler, Germany) and Nobel Biocare (NO, NobelActive, Internal RP 4.3 × 11.5 mm, Nobel Biocare Holding AG, Kloten, Switzerland). The implants with an internal angulated butt-joint connection were from Bego Semados (BE, BEGO Semados, S 4.1 × 11.5 mm, BEGO Implant Systems GmbH, Bremen, Germany). The geometrical properties of the investigated implant systems are listed in Table 1.

**Table 1:** Geometric design parameters of tested IACs. Abutment diameter was measured at most apical and coronal part of connection to depict different cone shapes

Abbreviation	Implant manufacturer	Implant diameter [mm]	Abutment diameter [mm]	Type of connection	Cone angle^a^ [°]	Cone length^b^ [mm]
MA	Medentika	3.5	2.8^c^ – 2.2^d^	Conical connection	10.5	0.78
MS	Medentis	4.1	3.0^c^ – 2.8^d^	Conical connection	11.3	0.92
NO	Nobel Biocare	4.3	3.3^c^ – 3^d^	Conical connection	12.2	0.74
BE	BEGO	4.1	2.8^c^ – 2.4^d^	Butt-joint connection	45	0.61

^a^ estimated from x-ray radiographs;

^b^ IAC height measured in radiography;

^c^ largest diameter of the abutment in the area of the mating zone

^d^ smalles diameter of the abutment in the area of the mating zone

For synchrotron radiography, pairs of implants and abutments were assembled and screw-tightened using the system-specific torque recommended by the manufacturers (MA, ME, BE: 30 Ncm, NO: 35 Ncm). According to ISO standard 14801:2008 [40], the implants were embedded in a 15-mm brass cylinder using a methylmethacrylate-based adhesive (X60, HBM Germany). The lower end of each implant was fixed to the test stand and a 10 mm steel ball was glued to the abutment for force application (X60, HBM) [11]. The implant abutment assemblies were positioned on a test stand and controlled forces of 30, 100 and 200 N (SH-500, PCE-group OHG, Germany) at an angle of 30 ° and 90 ° to the implant axis were applied. To evaluate unloaded dimensions of the IAC all samples were inspected prior to load application. Furthermore, microgap formation was assessed for three static loadings (30 N at 90°, 100 N at 90° and 200 N at 30°). For the visualization of residual abutment displacement after the final load stage (200 N at 90°) synchrotron microtomography was performed for one sample in addition to radiographic inspections.

### Synchrotron radiography and microtomography

Synchrotron phase-contrast radioscopy (PCR) was used to visualize gap formation at the BESSY-II light source (Helmholtz Center, Berlin) on the BAMline (wavelength: 24.8 pm; beam-height: 1.4 mm). In order to visualize the tangential extension of the microgap along the IAC as well as possible deformations at the implant shoulder, one Medentika (MA) implant (after loading) was scanned with microtomography (voxel size 4 μm) on the same beamline using the same energy and propagation distance. The latter refers to the distance between object and detector and enables PCR with strong edge-enhancement. X-ray images were therefore recorded with the detector (pixel sampling: 0.84 μm for the radiographs and 4 μm for micro-CT) placed 770 mm downstream if the measurement object. The microgap formation was evaluated by analyzing the phase contrasted fringes in the radiographies across the edge-enhanced IAC as described previously by Zabler et al. [32] The analysis uses numeric forward simulations of the optical Fresnel propagation and is able to detect microgaps down to 0.1 μm. Abutment dislocations were inspected at four different edges of the abutment trapezoid: “upper left“ (UL), “lower left“ (LL), “lower right“ (LR) and “upper right“ (UR). The loading always applied from the left-hand side in the images coordinates.

## Results

### Microgap formation

**Fig. 2:**
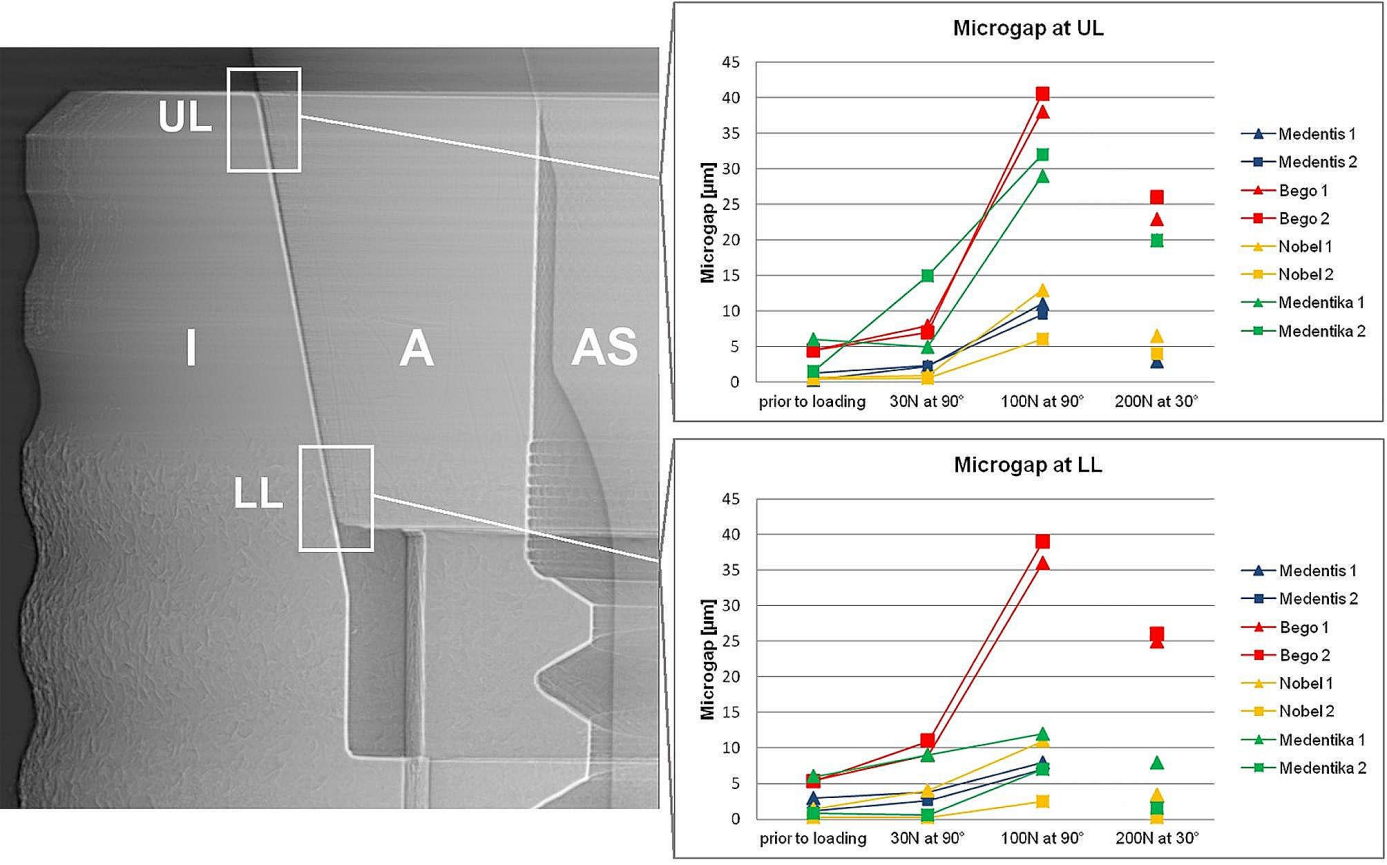
Microgap formation assessed using synchrotron radiography and phase-contrast radioscopy at upper (UL) and lower left abutment edge (LL) for different static load applications applied from left to right. Mounting force and loading angle induce increased microgaps at upper left abutment edge. (left radiographic image displays a MS implant as an example; I: Implant; A: Abutment; AS: Abutment screw)

**Fig. 3:**
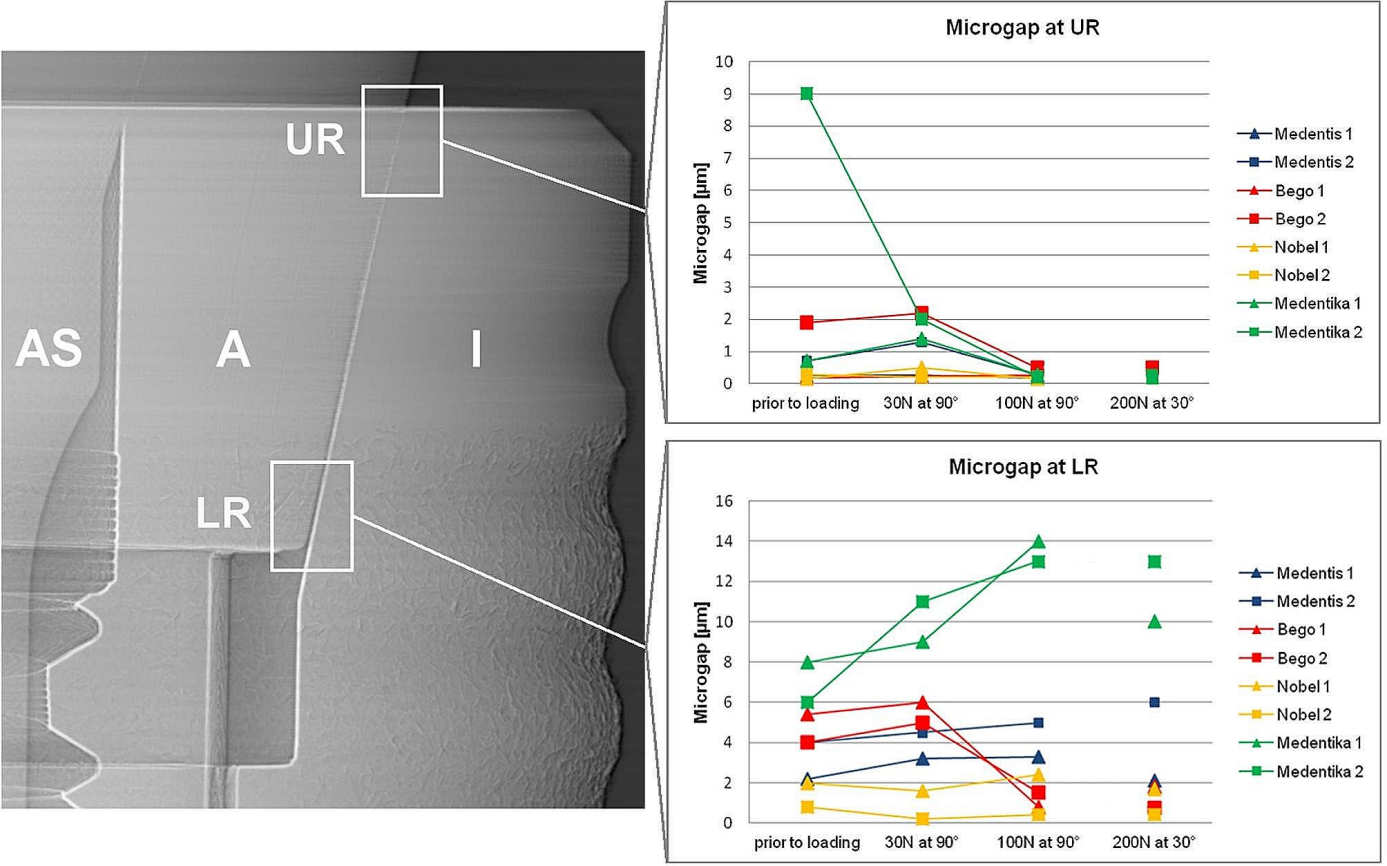
Microgap formation at upper (UR) and lower right abutment edge (LR) for different static load applications. All implant systems showed decreasing microgaps under incremental loading at upper right abutment edge (UR). (left radiographic image displays a MS implant as an example; I: Implant; A: Abutment; AS: Abutment screw)

Using synchrotron radiography the microgap was determined in all four systems in unloaded condition (0 N) and three different loading modes (30 N at 90°, 100 N at 90° and 200 N at 30°) (Table 2). Microgaps prior to loading were present in all systems varying from 0.2 to 9 μm (note that the smaller number is referring to the point where the microgap was most closed, generally the lower left (LL) corner, whereas the larger number refers to its widest opening, generally the upper left (UL) corner). Thereby the least microgap opening was found in NO implants (from 0.2 to 2 μm), whereas MA implants showed relatively large microgaps already prior to loading (from 0.7 to 9 μm) (Figs. 2 and 3).

**Table 2:** Microgaps of all implant systems under different loading conditions measured using synchrotron-based radiographies and phase-contrast radioscopy. (According to Zabler et al. an uncertainty of 50% can be assumed for microgaps up to 2 μm, whereas larger microgaps are determined with 2 μm uncertainty [31]).

		MS		BE		NO		MA	
	Position at IAC	1	2	1	2	1	2	1	2
Prior to loading	UL	0.3	1.3	4.5	4.5	0.7	0.5	6	1.5
	LL	3.0	1.2	5.5	5.3	1.4	0.2	6	0.8
	LR	2.2	4	5.4	4	2	0.8	8	6
	UR	0.3	0.7	0.2	1.9	0.2	0.3	0.7	9
30 N at 90°	UL	2.3	2.4	8	7	1	0.6	5	15
	LL	3.8	2.6	9	11	4	0.3	9	0.6
	LR	3.2	4.5	6	5	1.6	0.2	9	11
	UR	0.3	1.3	0.2	2.2	0.5	0.2	1.4	2
100 N at 90°	UL	11	9.5	38	40.5	13	6	29	32
	LL	8	7	36	39	11	2.5	12	7
	LR	3.3	5	0.8	1.5	2.4	0.4	14	13
	UR	0.2	0.3	0.3	0.5	0.2	0.2	0.3	0.2
200 N at 30°	UL	3	4.2	23	26	6.5	4	20	20
	LL	3.3	1.3	25	26	3.4	0.3	8	1.5
	LR	2.1	6	1.8	0.8	1.7	0.4	10	13
	UR	0.2	0.4	0.4	0.5	0.2	0.2	0.2	0.3

**Fig. 4:**
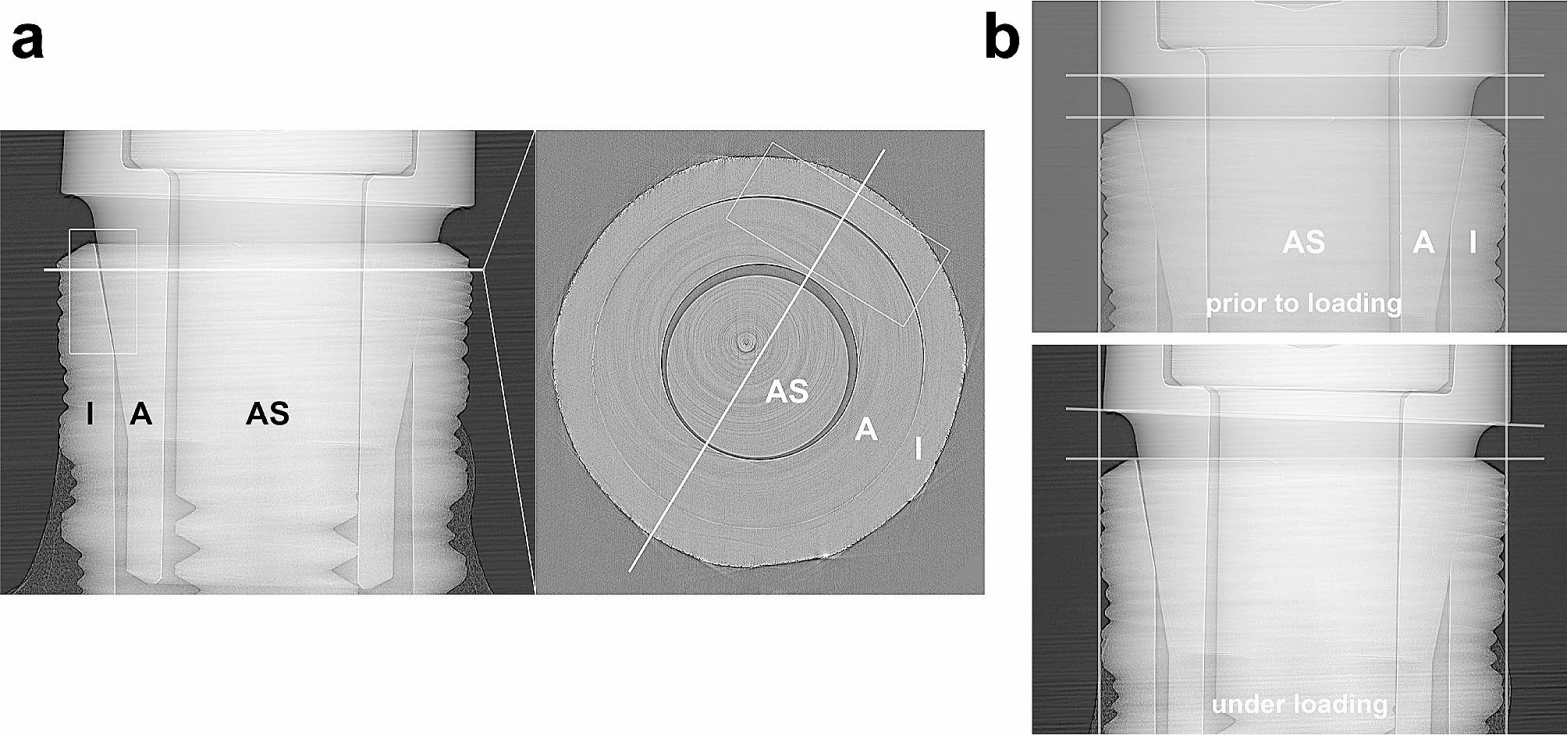
Synchrotron-based microtomography of microgap formation in MA implant. (I: Implant; A: Abutment; AS: Abutment screw); **a**: Even after force release a large microgap remains on the application side of the IAC. The azimuthal extension of the gap can be observed from horizontal micro-tomography slices (level of the slice is indicated by horizontal white line in the left hand tile, the diagonal white line in the right hand tile indicates the direction of previous load application) The side of application displays a sickle-shaped microgap (box in the right hand tile); **b**: Additional observation from radiography: off-axis loading leads to tilting abutment movement inside IAC and slight lateral implant wall deformation visible at right white positioning line

**Fig. 5:**
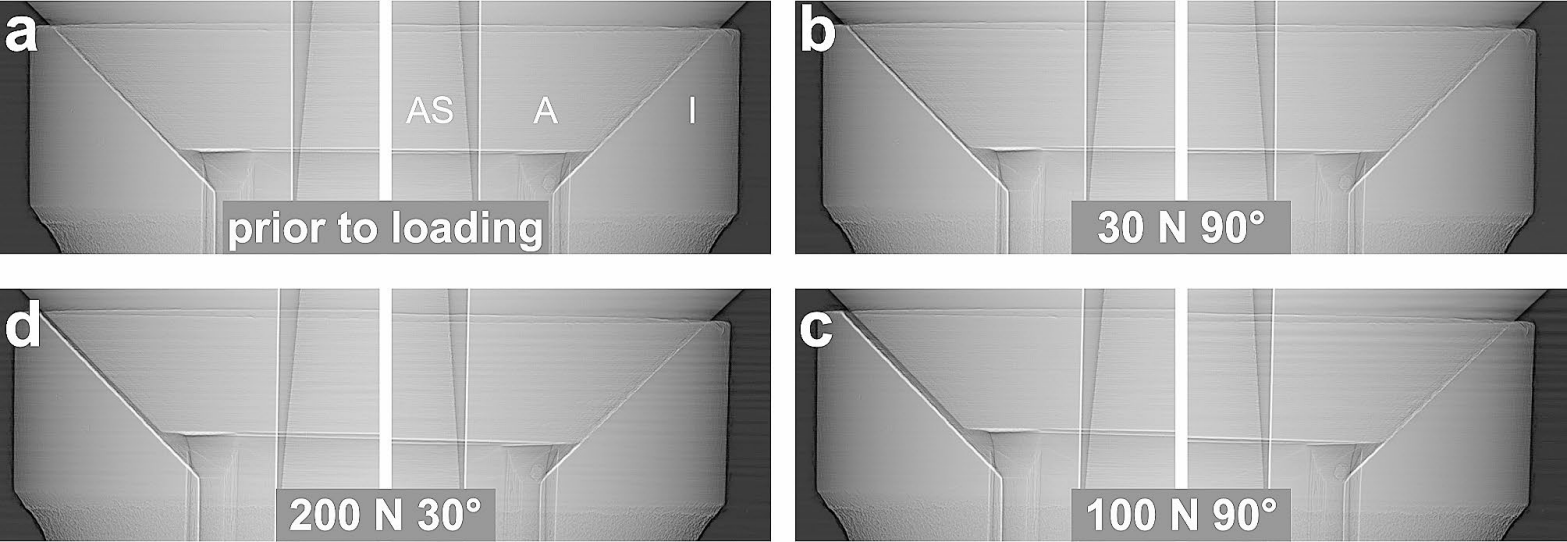
Phase-contrast radiographies of BE butt-joint connection prior to loading (**a**) and at force application of 30 N at 90° (**b**), 100 N at 90° (**c**) and 200 N at 30° (**d**) from the left side. Abutment displacement leads to almost parallel gap formation. Despite large microgaps (up to 40.5 μm) no implant wall deformation is visible on the opposing side indicated by transparent vertical positioning lines

Under horizontal application of 30 N force at (90º loading) the abutment was displaced relatively to the implant body resulting in a microgap opening at the coronal part of the IAC (UL) and in the most apical part of the IAC (LL) at the side of load application in all implants. MA implants thereby showed the widest opening (from 0.6 to 15 μm) followed by BE implants (from 0.2 to 11 μm). The microgap opening was most pronounced for a horizontal loading with a load application of 100 N (90º) for all implant systems. In this situation the microgap in all systems opened at the upper left (UL, ranging from 6 to 40.5 μm in all implants) and the lower left (LL, ranging from 2.5 to 39 μm) edges of the IAC, hence significantly wider than the unloaded state. The largest opening was observed in BE implants (from 0.5 to 40.5 μm). Additional to the microgap opening a tilting of the abutments with respect to the implant was observed in all systems under increasing force application. For systems with conical IACs the abutment dislocation with an opening of the microgap at UL and LL led to a canting of the abutment within the IAC on the opposite side. As a result, a closing of the microgap at the upper right abutment edge (UR) coincided with an opening at the most apical point (LR), as observed in MA, but also in other conical connections was assessed (Figs. 2 and 3). In MA implants, where this effect was pronounced, a visible deformation of the implant shoulder occurred as can be seen from reference lines (Fig. 4a and b). Microtomography of one MA implant revealed how the loading left such a dent as well as a permanent microgap after pressing the abutment against the implant wall with 200 N force. A different behavior was observed from the butt-joint connection (BE) which displayed a purely horizontal microgap opening at the load application side while closing the microgap on the coronal (UR) and apical abutment position (LR) on the opposite side (Figs. 1b and 5).

Note that two (presumably identical) implants were tested for each system under identical conditions. The intra system differences in microgap formation under load (Table 2) was in many cases higher than the uncertainty of the measurement. E.g. specimen 1 from the NO implants showed more than twice the opening (most pronounced in the LL corner) under all load conditions, compared to the second specimen of the same system which displayed significant less microgap formation.

## Discussion

The results of the present study demonstrate and visualize abutment movement and displacement in different conditions in implant systems with conical and butt-joint IACs. Applied synchrotron radiography confirm that microgaps were evident in all tested implants prior to loading and increased during load application. The examined implant abutment assemblies demonstrated microgaps ranging from 0.3 to 9 μm prior to loading. These findings are consistent with previous results using synchrotron-based radiography to investigate microgap formation in conical IACs [11, 12, 34, 35, 41]. Since a microgap is always present when two metal objects are joined, the microgap size is determined by the congruency of each metal part. A complete surface contact of machined parts i.e. implant and abutment is improbable due to the manufacturing process as the metal parts are processed using burrs leaving a specific surface texture [16]. Metal parts and their fabrication tolerances might vary depending on the precision of the manufacturing process executed [42]. In line with previous studies regarding the variation of fit and fabrication tolerance for different IACs the results demonstrate microgaps in the tested implant systems before and under loading [9, 43]. 

To date limited studies describe the influence of specific design parameters like cone angle, screw diameter, implant and abutment dimensions of the different implant systems on abutment displacement under off-axis loading [11]. To assess the effect of different cone angles on microgap formation Rack et al. investigated implants with a conical IAC ranging from 16° (Bone level) and 5.7° (Ankylos c/x, Ankylos Plus) using synchrotron-based radiography [34]. The authors concluded a higher resistance against off-axis forces with a 30° angle for greater cone angles because microgaps for these implants decreased. In the current study we could not assess a correlation between the cone angle and a higher resistance against loading in a 30 ° angle neither the extent of the microgaps evaluated. Still, the cone angle might be a crucial factor besides microgap formation for the mode of abutment displacement and implant shoulder deformation.

While the length of the mating zone does not appear to influence the degree of micromotion, it can determine the mode of microgap formation [34]. Despite a comparable length of the mating zone of the tested implants (ME 0.92 mm; MA 0.78 mm; NO 0.74 mm; BE 0.61 mm) small cone angles (MA 10.5 °; MS 11.3 °; NO 12.2 °) produced a V-shaped microgap at the loaded side, while connections with a greater cone angle (BE 45 °) performed an almost parallel gap opening (Figs. 1b, 4 and 5). Previous results testing conical connections with varying cone angles in a smaller range showed a tendency of this effect which is confirmed in the present study [11, 34]. Under load application the abutment annulling is stopped by reaching its contact with the implant wall at the side of load application determining the leverage point. The position of this contact is decisively influenced by the cone angle, the length of the mating zone, the internal length of the abutment and manufacturing tolerances between implant and abutment (Fig. 1). Conical connections with longer mating zones induce a contact at the lower area of the mating zone. This location can be altered by the angle of conus and the extension of the abutment into the implant. With shorter mating zones the contact with the implant wall can move below the mating zone which results in an increased lever arm. This can increase the torsion/lateral force on the counterlateral implant wall and as a consequence plastic deformation depending on the material thickness of the implant shoulder [12, 27, 44]. 

The deformation of the implant shoulder is associated with material fatigue as shown in previous studies using cyclic and static loading [11, 12, 34]. In implants with a butt-joint connection in which the interface of the abutment and the implant is either horizontal or slightly angulated (in BE 45°), applied force induces a movement of the abutment and a distribution of the force more vertically into the implant shoulder. Consequently, the design of the butt-joint connection induces less lateral force distribution on the implant shoulder when the adequate preload of the abutment screw is ensured and might result in less plastic deformation of the implant shoulder (Fig. 1b). Dittmer et al. stated that load bearing capacity and load to failure significantly differed in various IAC designs for load application in a 30° angle [28]. The authors assessed plastic deformation starting at lower loads (368–456 N) for implants with conical connections compared to butt-joint connections beginning at 891 N of load in non-fatigued implants. However, after cyclic loading mean values for all systems converged [45]. In accordance with these findings other studies reported advantages in failure strengths during dynamic loading for long internal tube-in-tube connections and deep joints compared to shorter internal or external connection designs [8, 28, 46]. The findings confirm an association between implant-abutment-connection design and wall deformation, corroborating the importance of plastic implant wall deformation in relation to peri-implant bone remodeling.

The present study investigated the abutment displacement of conical and butt-joint connections using synchrotron radiography. It is the first article to describe the mode and extent of microgap formation in butt-joint implant-abutment connections. Both mode and extent of microgap formation in butt-joint IAC seem to differ from conical connections. The results indicate that abutment displacement and implant wall deformation depend on the implant-abutment-connection design and the amount and angle of load applied.

## Conclusions

The present study allows further insights into the mode of abutment displacement under static off axis loading for different implant-abutment connection designs. The extent of microgap behavior under load application varied depending on the implant-abutment connection. Using an off-axis loading to simulate masticatory function the size of the microgap formation changed in all implant systems and increased with mounting load and angle applied. The mode and extent of microgap formation varied in conical and butt-joint connections. The micromovement of the abutment in some conical connections led to a plastic deformation of the implant wall under off axis loading that could induce a stress distribution in the crestal part of the peri-implant bone. Since several studies proposed peri-implant bone loss due to overloading under appropriate conditions such as peri-implant inflammation, these findings are of high clinical relevance.

## Data Availability

The datasets used and/or analyzed during the current study available from the corresponding author on reasonable request.

## Abbreviations

BE: BEGO Semados implants
IAC: Implant Abutment connection
LL: Lower left abutment position
LR: Lower right abutment position
MA: Medentika implants
MS: Medentis ICX implants
NO: Nobel Biocare implants
PCR: Phase Contrast radioscopy
SRX: Synchrotron Based radiation
UL: Upper left abutment position
UR: Upper right abutment position
